# A Method to Estimate the Efficacy vs. Effectiveness in Meta-Analysis of Clinical Trials with Different Adherence Scenarios: A Monte Carlo Simulation Study in Nutrition

**DOI:** 10.3390/nu13072352

**Published:** 2021-07-09

**Authors:** Miguel Ángel López-Espinoza, José Antonio Lozano-Lozano, David Prieto-Merino

**Affiliations:** 1 Health Science Department, Universidad Católica de Murcia (UCAM), 30107 Murcia, Spain; 2 Instituto de Ciencias Biomédicas, Universidad Autónoma de Chile (Chile), Santiago 7500912, Región Metropolitana, Chile; jose.lozano@uautonoma.cl; 3 Cátedra Internacional de Análisis Estadístico y Big Data, Universidad Católica de Murcia (UCAM), 30107 Murcia, Spain; dprieto@ucam.edu

**Keywords:** fruits and vegetables, intention to treat, meta-analysis, Monte Carlo method, SBP, DBP, LDL, HDL

## Abstract

Randomized clinical trials (RCTs) evaluating the effectiveness of interventions to promote fruit and vegetable (FV) consumption usually report intention-to-treat (ITT) analysis as the main outcome. These analyses compare the randomly assigned groups and accept that some individuals may not follow the recommendations received in their group. The ITT analysis is useful to quantify the global effect of promoting the consumption of FV in a population (effectiveness) but, if non-adherence is significant in the RCT, they cannot estimate the specific effect in the individuals that increased their FV consumption (efficacy). To calculate the efficacy of FV consumption, a per protocol analysis (PP) would have to be carried out, in which groups of individuals are compared according to their actual adherence to FV consumption, regardless of the group to which they were assigned; unfortunately, many RCTs do not report the PP analysis. The objective of this article is to apply a new method to estimate the efficacy of Meta-analysis (MA) PP which include RCTs of effectiveness by ITT, without estimates of adherence. The method is based on generating Monte Carlo simulations of percentages of adherence in each allocation group from prior distributions informed by expert knowledge. We illustrate the method reanalyzing a Cochrane Systematic Review (SR) of RCTs on increased FV consumption reported with ITT, simulating 1000 times the estimation of a PP meta-analyses, and obtaining means and ranges of the potential PP effects. In some cases, the range of estimated PP effects was clearly more favourable than the effect calculated with the original ITT assumption, and therefore this corrected analysis must be considered when estimating the true effect of the consumption of a certain food.

## 1. Introduction

From the theoretical models published by Harman in 1965 for the case of antioxidants [1], such as those of Trowell and Burkitt (separately) who, between 1972 and 1985, proposed the fiber hypothesis [2], the group of fruits and vegetables (FV) has gained a good reputation for its bioactive components [3,4,5,6,7,8,9] antioxidants [10], as their long-term intake is associated with improved cardiovascular health [11].

To support this scenario, there are MA of systematic reviews (SR) of randomized clinical trials (RCTs), which are catalogued as the best available evidence to evaluate the outcome of an intervention [12,13]. In turn, RCTs are generally analyzed by intention to treat (ITT), a principle established in the 1960s, accepted by the scientific community [14], and required in RCT methodologies [15] and systematic reviews [16]. It is based on analyzing all the individuals in the group to which they were randomly assigned [14,17,18,19], regardless of whether or not they adhered to the assigned intervention [20]. This safeguards the usefulness of random assignment [21,22], whose main function is to eliminate known or unknown initial confounding factors [21]; protects against attrition bias [23]; and, thus, allows the intervention of the patient to be interpreted as a causal effect trial [24,25].

ITT is based on the idea that the effectiveness of a treatment is the result of three factors: (a) the efficacy of the active principle or intrinsic characteristic of the intervention, (b) the clinician’s ability to persuade and manage the application, and (c) the ability of the patient to adhere to or follow the treatment indications [26], thus reflecting a more realistic scenario of the population that intends to benefit, as it admits aspects such as non-compliance and deviations from the protocol [27]. Therefore, what ITT really assesses is the effectiveness of the assigned recommendation, not the efficacy of the administered intervention itself [28], especially if the clinical utility of the intervention is to be estimated. Thus, ITT is appropriate for studies which aim to evaluate a treatment policy [29], such as a change in dietary habit by recommendation of intake.

When there is a lack of adherence, an ITT analysis always tends to estimate more similar results between the RCT groups. Therefore, it is likely that the MA of RCTs to increase FV consumption analyzed by ITT will underestimate the true effect (efficacy) on health outcomes of increasing FV consumption [30,31], and this may be contributing to some inconsistent results seen in the field of nutritional epidemiology [32]. To better approximate the efficacy of FV consumption, a per protocol analysis (PP) would need to be done, comparing groups of individuals according to their actual adherence to FV consumption regardless of the group to which they were initially randomized. Although many RCTs do not report the percentages of adherence of the participants to the recommendations received, we can recalculate the PP analyses from the ITT analyses if we assume percentages of adherence to the intervention in each allocation group.

We propose a Monte Carlo (MC) simulation method which attempts to compensate for the possible lack of adherence in RCTs and to obtain a more realistic estimate of the effect of increased FV consumption, by means of a PP analysis based on the ITT analyses reported, and assuming a distribution of potential percentages of adherence to the recommendations in each arm of the trial. Thus, we hypothesize that proposed new estimation method to recalculate effect sizes in meta-analysis with simulated adherence percentages will show smaller type I errors compared to the original ones which included RCT by ITT.

Our aim here is not to analyze, discuss, or understand the reasons for lack of adherence, or the strategies to improve adherence. The contribution of this work is to develop a method to estimate the efficacy of a nutritional intervention when lack of adherence is a concern, and it has not been properly measured but can be approximately guessed with expert knowledge. To the best of our knowledge, there is no method that is routinely applied in the analysis of nutritional interventions to address this potentially important bias. Here, we present a method and show how to use it in a real dataset.

## 2. Materials and Methods

We first present the algorithm to do a per protocol estimation of a meta-analysis which was originally estimated by ITT, and where we do not have information of true adherence on each arm of each RCT. Then, we will apply our method to a real dataset as an example.

### 2.1. Deduction of Statistics per Protocol from Those Reported by ITT

Notation: Let, for an RCT, be $N_{I}= [n_{i},n_{c}]$ the vector of sample sizes analyzed in each group by intention to treat (with *I* = subscript referring to intention to treat, *i* = subscript of the intervention group, and *c* = subscript of the control group), and let $M_{I}= [m_{i},m_{c}]$ the vector of means of the outcome variable of interest, and $V_{I}= [v_{i},v_{c}]$ the vector of variances in those groups, therefore *m_i_–m_c_* being the estimated ITT effect of the trial (effectiveness). Let *p_i_* be the percentage of participants assigned to the intervention group who really adhere to the recommendations received (and therefore a 1 − *p_i_* percentage of patients assigned to the intervention group that do not adhere), and let *p_c_* be the percentage of patients assigned to the control group who also adhere to the consumption of FV, while the rest (1 − *p_c_*) do not adhere. Let $N_{P}= [n_{1},n_{0}]$ be the vector of sample sizes per protocol (with *P* = subscript referring to per protocol analysis, 1 = subscript for group of patients who adhere to the recommendations of the intervention, 0 = subscript for group of patients who do not adhere). Let $M_{P}= [m_{1},m_{0}]$ be the vector of outcome means, and $V_{P}= [m_{1},m_{0}]$ the vector of variances in those groups per protocol, therefore *m_i_*–*m_c_* being the estimated PP effect of the trial (efficacy). We are going to show how we can deduce, for an RCT, the unreported vectors $N_{P},\;M_{P},\;V_{P}$ from the reported vectors $N_{I},\;M_{I},\;V_{I}$ assuming adherence proportions $p_{i},\;p_{c}$.

In these analyses, we will assume that the means and variances of the outcome in the adherent individuals is independent of whether those were initially assigned to the control group or the intervention group, as what determines the outcome is the actual consumption, and not the initial assignment. We will assume something analogous for the mean and variance of the non-adherent individuals. All vectors will from now on be considered in column matrix notations. 

Sizes in adherence groups: The number of adherent individuals is the sum of the adherent among those who were assigned to the intervention (*n_i1_*) plus the adherents in those who were assigned to the control group (*n_c1_*). These quantities can be estimated by multiplying the adherence proportions by the sample sizes in each group. The number of non-adherent individuals can be estimated by difference from the total study size.

(1) $$n_{1}=n_{i1}+n_{c1}=n_{i}p_{i}+n_{c}p_{c}\;\;\left(“\mathrm{for}\;\mathrm{adherent}\;\mathrm{individuals}”\right)\;n_{0}=n-n_{1}\;\;\left(“\mathrm{for}\;\mathrm{non}-\mathrm{adherent}\;\mathrm{individuals}”\right)$$

Real outcome averages: The arithmetic mean of the outcome observed in subjects assigned to the intervention group must be the sum of the mean outcome in subjects who actually consumed FV (*m*_1_), multiplied by the proportion of adherence in the intervention group *p_i_*, and the mean outcome in subjects who did not consume FV (*m*_0_), multiplied by the proportion of non-adherents in the intervention group (1 − *p_i_*).

(2) $$m_{i}=m_{1}p_{i}+m_{0}\left(1-p_{i}\right)$$

Similarly in the control group we can deduce that:

(3) $$m_{c}=m_{1}p_{c}+m_{0}\left(1-p_{c}\right)$$

Putting the two previous expressions in matrix form we obtain:

(4) $$[\begin{array}{c} m_{i}\\ m_{c} \end{array}]= [\begin{array}{cc} p_{i} & \left(1-p_{i}\right)\\ p_{c} & \left(1-p_{c}\right) \end{array}] [\begin{array}{c} m_{1}\\ m_{0} \end{array}]$$

Using vector notation, and calling A the matrix of proportions of adherence, we can write Equation (2) as $M_{I}=A\cdot M_{P}$ and then:

(5) $$M_{P}=A^{-1}\cdot M_{I}$$

Real outcome variances: In each assignment group we can express the observed variance as a weighted mean of the variance in those who are adherent and those who are not. For example, in the intervention group, the variance of the effect would be calculated:

(6) $$v_{i}=\frac{\sum_{j=1}^{n_{i}}x_{ji}^{2}}{n_{i}}-m_{i}^{2}$$

where *x_ji_* is the “outcome” experienced by individual *j* in the intervention group. This sum can be decomposed into the outcomes of those individuals who were adherents in the intervention group and those who were not, leaving the following expression:

(7) $$v_{i}=\frac{\sum_{j=1}^{n_{i1}}x_{ji1}^{2}}{n_{i1}}\frac{n_{i1}}{n_{i}}+\frac{\sum_{j=1}^{n_{i0}}x_{ji0}^{2}}{n_{i0}}\frac{n_{i0}}{n_{i}}-m_{i}^{2}=\frac{\sum_{j=1}^{n_{i1}}x_{ji1}^{2}}{n_{i1}}p_{i}+\frac{\sum_{j=1}^{n_{i0}}x_{ji0}^{2}}{n_{i0}}\left(1-p_{i}\right)-m_{i}^{2}$$

where *x_ji1_* and *x_ji0_* are the outcomes on the adherent and non-adherent individuals in the intervention group, respectively. Now, using an expression analogous to Equation (6), and assuming that the outcomes are determined by adherence and not by the initial assignment, the sum of the squared of a subgroup of adherents can be related to the variance *v_1_* and the average expected in adherents, in general:

(8) $$\frac{\sum_{j=1}^{n_{i1}}x_{ji1}^{2}}{n_{i1}}=v_{1}+m_{1}^{2}\;\;\;\;\;\;\mathrm{and}\;\;\;\;\;\frac{\sum_{j=1}^{n_{i0}}x_{ji0}^{2}}{n_{i0}}=v_{0}+m_{0}^{2}$$

Additionally, using the expressions of (8) in Equation (7) we would have:

(9) $$v_{i}=\left(v_{1}+m_{1}^{2}\right)p_{i}+\left(v_{0}+m_{0}^{2}\right)\left(1-p_{i}\right)-m_{i}^{2}$$

Taking into account that for the control group there would be an analogous expression, we can write both expressions together using matrices:

(10) $$[\begin{array}{c} v_{i}\\ v_{c} \end{array}]= [\begin{array}{cc} p_{i} & \left(1-p_{i}\right)\\ p_{c} & \left(1-p_{c}\right) \end{array}] [\begin{array}{c} v_{1}+m_{1}^{2}\\ v_{0}+m_{0}^{2} \end{array}]- [\begin{array}{c} m_{i}^{2}\\ m_{c}^{2} \end{array}]$$

Additionally, calling $M_{P}^{2}= [\begin{array}{c} m_{1}^{2}\\ m_{0}^{2} \end{array}]$ and $M_{I}^{2}= [\begin{array}{c} m_{i}^{2}\\ m_{c}^{2} \end{array}]$, we can rewrite the expressions in (10):

(11) $$V_{I}=A\cdot [V_{P}+M_{P}^{2}]-M_{I}^{2}\;V_{I}+M_{I}^{2}=A\cdot [V_{P}+M_{P}^{2}]\;V_{P}=A^{-1}\cdot [V_{I}+M_{I}^{2}]-M_{P}^{2}$$

### 2.2. Simulation of an MA Per Protocol

If the real percentages of adherence in each arm of each RCT were known $[p_{i},\;p_{c}]$, we could directly estimate the per protocol statistics by applying the formula in the previous section and reconducting the MA, producing a global estimated efficacy with its 95% confidence interval. However, as these percentages are unknown, we are going to simulate them randomly from an a priori distribution. To reflect the uncertainty about the simulated percentages in this MA result, we will repeat the process of simulating percentages and calculating the MA 1000 times. We will thus have 1000 MA estimates per protocol with as many confidence intervals. We can finally calculate an average effect of all these iterations with a confidence interval which includes both the uncertainty due to random error, and the uncertainty due to the simulation of the adherence percentages.

For each of the 1000 iterations we follow the following process:

Firstly for each RCT, the adherence percentages for the intervention and control groups were simulated $[p_{i}^{*},\;p_{c}^{*}]$ sampling each one using a beta distribution, $\beta_{\left(a,b\right)}$ where *a* = 100 × *p_i_*, *b* = 100 (1 − *p_i_*) and *p_i_* is an a priori expected adherence. For example, if, a priori, we expected the proportion of adherents among those in the intervention group to be 95% (p=0.95) then we would sample the proportion for the equations of a distribution $p_{i}\sim \beta_{\left(95,5\right)}$;

Secondly for each RCT, statistics per protocol are calculated $N_{P},\;M_{P},\;V_{P}$, applying the formulas of the previous section on the reported statistics $N_{I},\;M_{I},\;V_{I}$;

Finally, the MA of the RCTs is achieved with the statistics per protocol with the same method as the original MA of Hartley L et al., 2013 [33] and a PP effect mean is obtained with its corresponding confidence interval and a *p*-value. (Supplementary Materials Computer Code S1).

After 1000 iterations of the three steps above, 1000 confidence intervals and 1000 *p*-values are obtained for the PP effect. We can obtain a global PP mean effect and a global 95% interval by repeatedly sampling from the 1000 simulated confidence intervals. We can also calculate a mean of the *p*-values and make a 95% interval of their distribution. Finally, we can calculate the percentage of simulations that had a *p*-value < 0.05, according to the expression:

(12) $$Prop\;(p_{value}<0.05)=\frac{\sum simulations\;with\;p_{value}<0.05}{total\;simulations}\cdot 100$$

We repeat everything for different expected values of adherence in each group *p_i_* of 0.70, 0.80, 0.90, and 0.95, combined with expected values of *p_c_* of 0.1, 0.2, and 0.3, to study different adherence situations. In turn, in each scenario, we calculated the MA for four different variables reported in the original article: SBP, DBP, LDL cholesterol, and HDL cholesterol.

For all the calculations, we used the statistical software R version 4.0.0 [34] with the user interface R-Studio [35] and the *metafor* package [36].

**Figure 1:**
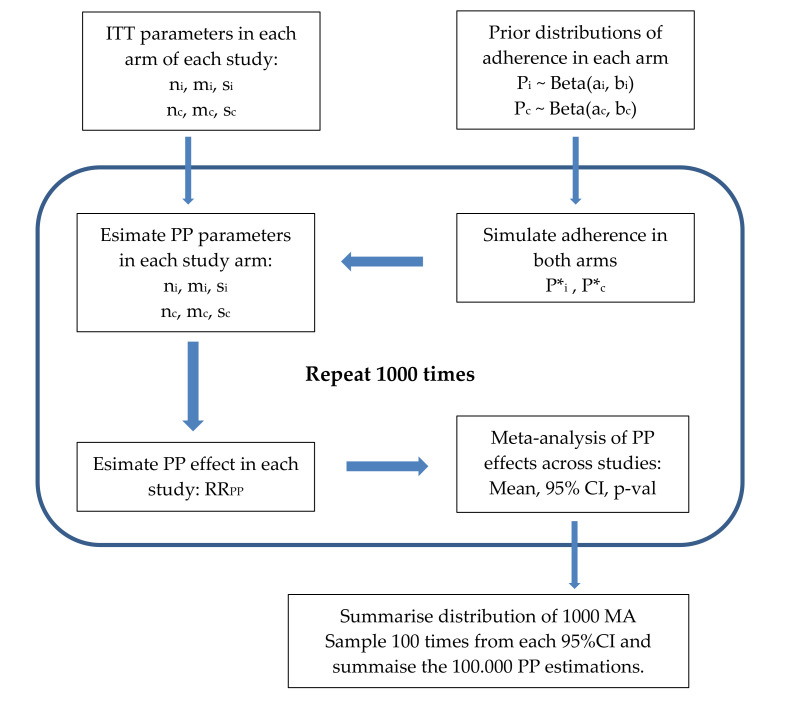
Flow chart of algorithm.

A flow chart of the process is presented in Figure 1.

### 2.3. Data

To illustrate our method, we reanalyzed a previously published meta-analysis that estimated the effectiveness of increasing FV intake on cardiovascular variables, based on RCTs that report ITT results.

We have used data from the SR published by Hartley L et al., 2013 [33] available in the Cochrane Library. This SR has been used so far in Guidelines for preventive activities in general practice, 9th edition, published in 2016, plus its updated version, published in 2018. This SR estimated the effectiveness of the recommendations for FV consumption for primary prevention of cardiovascular diseases (CVD) comparing two arms: intervened group versus control. The RCTs include only subjects older than 18 years, both from the general population or with cardiovascular risk factors (smokers, dyslipidaemia, and arterial hypertension). Studies were excluded where more than 25% of the participants had previously experienced a cardiovascular event (myocardial infarction, cerebrovascular accident, revascularization procedure, angina or coronary artery disease, or cerebrovascular accident) or who had been diagnosed with type 2 diabetes mellitus, as these are factors that increase the risk of cardiovascular event. The interventions lasted at least three months, and were based on the action of recommending entire FV, that is, interventions based on the recommendation of juices and multifactorial interventions were excluded to avoid confusion. All studies presented a comparison group, based on usual diet or with minimal intervention (for example, diet brochures) that did not involve a direct boost to take FV. From this SR, four MA were completed on four different outcomes: systolic blood pressure (SBP), diastolic blood pressure (DBP), LDL cholesterol, and HDL, with studies that declared having applied ITT. We applied our analysis to the four different outcome variables reported in the original article.

## 3. Results

**Figure 2:**
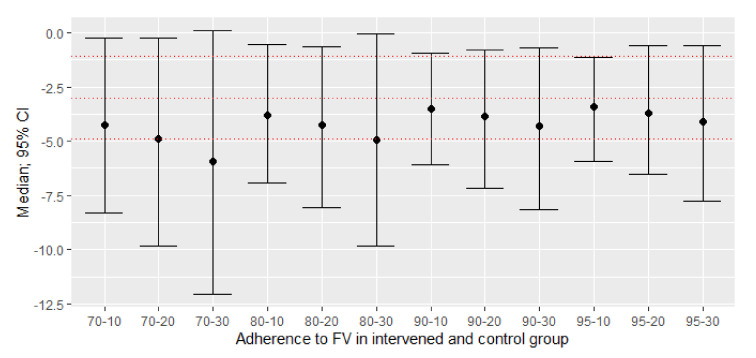
New effect sizes: difference between adherent and non-adherent participants of means of their post-pre changes of SBP (mmHg) with different scenarios of non-adherence to consumption. The mean difference with a confidence interval (95%) of 1000 simulations. The red dashed lines represent the mean with a 95% confidence interval of the original ITT meta-analysis. All point estimates were lower than the mean of the original study.

With the original ITT MA, the recommendation to consume FV achieves a statistically significant mean reduction of −3 mmHg in SBP (95% CI: −4.92; −1.09) compared to the control group, based on 444 intervened subjects and 447 controls, in two studies, with I^2^ = 0%. After repeating the PP MA analysis 1000 times, the global means in all scenarios show more obvious mean differences in favor of the adherent group (Table 1), but with wider confidence intervals than the ITT analysis that are wider, as the lack of adherence to the randomized group increases (Figure 2).

### 3.1. MA for Diastolic Blood Pressure (DBP)

**Figure 3:**
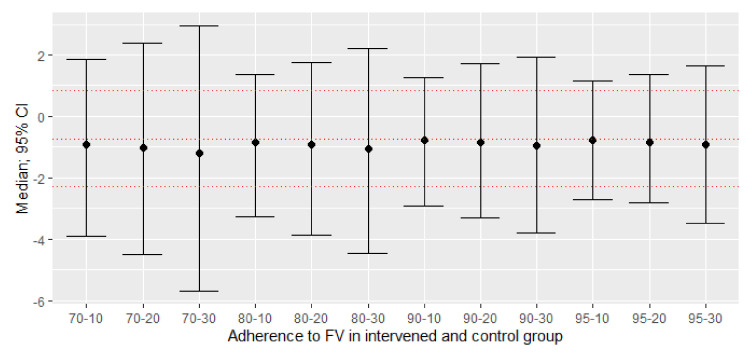
New effect sizes: difference between adherent and non-adherent participants of means of their post-pre changes of DBP (mmHg) with different scenarios of non-adherence to consumption. The mean difference with a confidence interval (95%) of 1000 simulations. The red dashed lines represent the mean with a 95% confidence interval of the original study. Although all the estimates were lower than the null value and the original study, they failed to achieve *p* < 0.05.

Regarding DBP, in the ITT meta-analysis a mean difference of −0.74 (95% CI: −0.30; +0.83), *p* = 0.3580 was reported. Now, with different adherence scenarios, the mean differences ranged between −0.79 and −1.22 mmHg of DBP, none of them being statistically significant (Table 2 and Figure 3).

### 3.2. MA for Low Density Lipoproteins

The original ITT MA estimated a mean difference of −0.17 (95% CI: −0.38; +0.03) in LDL, with *p* = 0.1028 (I^2^ = 0%). In the simulated PP analyses, statistically significant decreases in LDL were observed, except in some scenarios, as indicated in Table 3.

**Figure 4:**
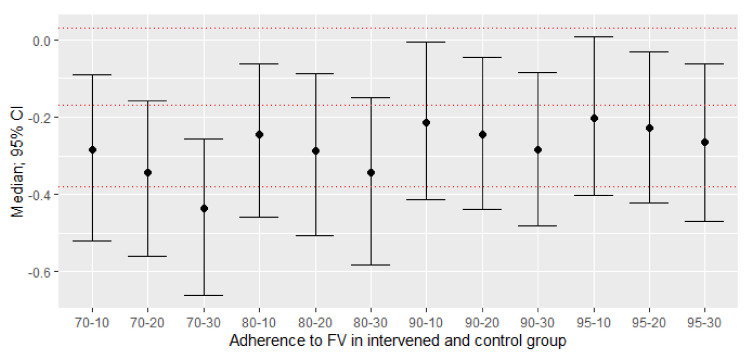
New effect sizes: difference between adherent and non-adherent participants of means difference with a confidence interval (95%) of 1000 simulations. The red dashed lines represent the mean with a 95% confidence interval of the original study. All point estimates were lower compared to the mean of the original study, and their confidence intervals are below the null value, with *p* < 0.05.

Figure 4 shows that not following the recommendation assigned in the intervened and control group, with proportions of 0.30 (1−0.70) and 0.20, respectively, allowed us to obtain the most favorable effect sizes towards the reduction in LDL; however, all the scenarios presented significant decreases in LDL (Figure 4).

### 3.3. MA for High Density Lipoproteins

**Figure 5:**
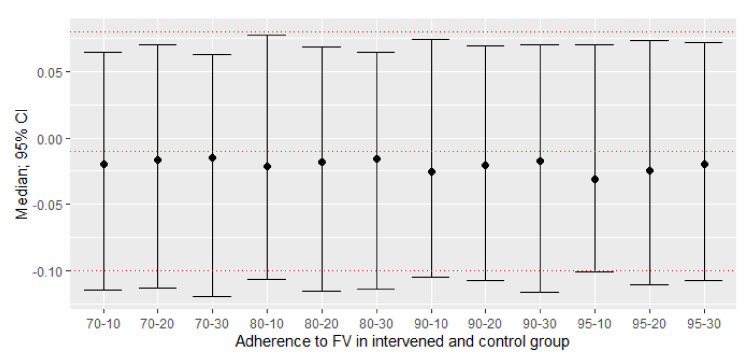
New effect sizes: difference between adherent and non-adherent participants of means of their post-pre changes of HDL (mmol/L) with different scenarios of non-adherence to consumption. The mean difference with a confidence interval (95%) of 1000 simulations. The red dashed lines represent the mean with a 95% confidence interval of the original study. All point estimates and their respective confidence intervals were similar compared to the mean of the original study, without observing statistically significant effects.

The original ITT analysis showed no significant effect on HDL, and neither did the simulated PP analyses (Table 4). Indeed, it can be observed in Figure 5 that none of the simulated values in the evaluated scenarios presented statistically significant effects and, furthermore, they were all close to the original value.

## 4. Discussion

The present study evaluated the impact on the estimation of the efficacy of FV consumption of 12 scenarios that simulated different deviations from the assigned recommendation of FV consumption in an MA, finding some new effect sizes on cardiovascular variables that are far from the null value.

Regarding SBP, as the assumed percentages of non-adherence increase in the intervention and control groups, the estimated PP effect size tends to increase (lower SBP in the intervention group), although also to the detriment of percentage with *p* < 0.05. This is possibly due to increased heterogeneity between the recalculated effects of RCTs containing different simulated adherence percentages, with the consequent widening of the confidence intervals. This heterogeneity increased further when the percentage of non -adherence in the control group was high (30%), giving more room to sample distant values non-adherence between studies. 

Adherence could be explained by a variety of factors, such as: (a) the type and duration of nutritional treatment received [37,38], highlighting the importance of follow-up to assess the efficacy of the intervention in the short and long term [39,40]; (b) motivation: as one of the facilitating factors in adherence to FV consumption [41,42], versus who was assigned to the control group; (c) access to information: adherence may be hampered due to the fact that practical aspects of the assigned intervention are unknown, such as portion sizes and the need for food variability [43]; (d) employment status and occupation: occupation gathers information on life styles and conditions related to education and income level; at a general level, a better professional qualification provides better working conditions and higher income, conditions associated with a higher prevalence of FV consumption [44,45]; (e) psychosocial stress: subjects with a history of cardiovascular disease undergoing a nutritional intervention [46], such as the consumption of FV according to recommendations assigned by the professional, in the long term generate allostatic load, that is, a maladaptive response, in this case not following the recommendations indicated [47]; (g) compensatory health beliefs: a factor that can influence adherence to FV consumption and consists of the belief that unhealthy behavior can be compensated; for example, eating unhealthily can be offset by exercising [42]; or, finally, (h) psychotherapeutic interventions: among psychotherapeutic interventions for modifying behaviors to promote adherence to nutritional interventions [48], we find Behavioral Activation (BA), focused on the reduction in avoidance behaviors and the development of routines and rewarding behaviors that allow greater adherence to FV consumption in adults with subsyndromal symptoms of depression [49]. 

In the case of LDL, it is interesting how the evidence progressed from an effect of −0.17 with a *p*-value = 0.1028 in the ITT analysis, to presenting larger effect sizes with percentages of simulations with *p* < 0.05 observed in a large percentage of simulated cases; this is possibly due to the fact that the *p*-value of the original study was relatively close to the statistically significant value, added to the fact that I^2^ with recalculated values due to the random inclusion of adherence percentages remained at the same value than the original study, also avoiding presenting confidence intervals that have crossed the null value. This finding with PP reinforces the idea that dietary fiber [37,38,39] and some flavonoids [42,43] that contribute FV can lower LDL, and the incongruity with the ITT results exemplifies the difference between effectiveness and efficacy with PP analysis, capturing what might happen to a person that actually increases their FV consumption, while the ITT analysis is a more conservative estimate of the net effect of the intervention in the population.

In HDL, the high *p*-value of the original study did not allow the efficacy recalculations with the inclusion of adherence percentages to be significant. This denotes that the coincidence between both analyses does not always ensure a joint conclusion of effectiveness (in real life) and efficacy (of its active principle), as they must be used for that which they are intended. However, it is important to establish, as a general pattern, that the results with PP analysis provide more extreme effects than ITT, regardless of whether the latter’s estimates are statistically significant. 

This study is not without limitations, among which it stands out that the Systematic Review used here has only two RCTs that exclusively examine the effects of the advice to increase consumption of FV for the primary prevention of CVD. This situation is not infrequent in SR of health interventions, though it is more so if we intend to combine RCTs that must go through rigorous stages of planning, permits, application, and measurement of variables. A study which examined 22,453 SRs from the Cochrane database found that the median was 3 studies per MA, with an interquartile range of 2 to 6, and 36% included only 2 studies [50]. This implied that our method was not applied in mixed effects meta-analysis, and therefore it was not possible to analyze what would be the impact of potential moderators on the effect size with the different simulated adherence scenarios. However, in future studies that find a meta-analysis with a greater number of studies by ITT, we may analyze how the adherence scenarios may change in a context of adjustment with confounding factors.

Our model (as with every model) must make assumptions to simplify the mechanisms of nature in such a way that the model is understandable and manageable by a human. Some of those assumptions might be over simplistic, and there is always a trade-off between simplicity and complexity. One limitation is that we have assumed and simulated a common level of adherence in all studies in the meta-analysis, but adherence might vary greatly between studies. Our model could be easily modified to simulate adherence levels separately for each study using different prior distributions. We have not shown that possibility here, but it will be present in our next refinement of the model. 

Another simplification in our model is that we do not consider patient losses. Furthermore, adherence relapses were not considered (i.e., a patient who deviates from the assigned intervention might return to it after a while). Accounting for this in the models would require thinking of the gaps where the patients were not adherent, which might depend on the duration of the interventions. We would have multiple simulation scenarios that escape the scope of this article.

Another limitation is that adherence was only simulated here in a dichotomous way, i.e., the participant is either fully adherent or non-adherent. This is not realistic; there could be different degrees of adherence and the efficacy might also depend on the level of adherence. It would be possible to incorporate this complexity of adherence patterns, but it would require a more complex multinomial a priori distribution true adherence in each trial arm.

Finally, one could potentially test whether some assumptions of our model are true or false. For example, we assume that the efficacy of the intervention is the same in all individuals that were adherent to the intervention, regardless of the group where they were initially allocated, but this might not be correct. Efficacy might depend on patient characteristics, and these might, on average, differ between adherent patients initially allocated to different study arms. However, to test this assumption we would need data from studies where they had estimated the true proportion of adherence and the efficacy in each group, and this can vary from study to study. We see this kind of validation as an interesting next step and an opportunity for refining the model, maybe in different sub fields of the nutrition research separately.

## 5. Conclusions

In conclusion, incorporating an estimated adherence to a PP analysis of RCTs in nutrition can produce effect size estimations different from those estimated by ITT, and therefore it is a variable that must be considered if one wants to estimate the true effect of adhering to the intervention (efficacy), rather than the effect of recommending consumption in the population (effectiveness).

We would recommend that researchers who analyze the effects of nutritional interventions routinely apply this kind of sensitivity analysis to give a clearer picture of the situation and estimate the possible difference between the efficacy and the effectiveness of the interventions. 

## Figures and Tables

**Table 1:** Impact of different adherence scenarios of consuming FV on SBP.

Adherence		n_1_ *	n_0_ *	Difference **	*p*-Value *	I^2^ *	Prop of *p* < 0.05
$p_{i}$	$p_{c}$						
0.70	0.10	356 (317; 394)	535 (497; 574)	−4.25 (−8.33; −0.26)	0.0266 (0.0027; 0.0727)	57.25 (29.35; 75.73)	85.1
	0.20	400 (362; 441)	491 (450; 529)	−4.91 (−9.85; −0.26)	0.0380 (0.0033; 0.1070)	70.36 (44.27; 86.18)	68.1
	0.30	446 (400; 488)	445 (403; 491)	−5.91 (−12.04; 0.09)	0.0453 (0.0009; 0.1358)	80.24 (48.77; 93.21)	55.5
0.80	0.10	400 (365; 437)	491 (454; 526)	−3.82 (−6.92; −0.56)	0.0153 (0.0016; 0.0515)	41.72 (16.83; 63.85)	97.3
	0.20	444 (407; 485)	447 (406; 484)	−4.25 (−8.06; −0.66)	0.0257 (0.0023; 0.0806)	56.06 (27.58; 76.91)	85.7
	0.30	489 (448; 532)	402 (359; 443)	−4.92 (−9.84; −0.05)	0.0365 (0.0027; 0.1035)	68.60 (40.57; 85.51)	69.5
0.90	0.10	444 (414; 474)	447 (417; 177)	−3.52 (−6.08; −0.96)	0.0059 (0.0004; 0.0281)	22.66 (0; 45.32)	99.9
	0.20	488.5 (455; 523)	402.5 (368; 436)	−3.84 (−7.14; −0.78)	0.0139 (0.0007; 0.0503)	38.55 (10.13; 61.45)	97.3
	0.30	533.5 (496; 571)	357.5 (320; 395)	−4.31 (−8.14; −0.70)	0.0234 (0.0012; 0.0735)	52.58 (20.76; 73.54)	87.9
0.95	0.10	467 (441; 493)	424 (398; 450)	−3.52 (−5.92; −1.13)	0.0003 (0.0001; 0.0007)	<0.0001 (<0.0001; 0.0002)	100
	0.20	512 (480; 544)	379 (347; 411)	−3.70 (−6.53; −0.62)	0.0086 (0.0004; 0.0366)	28.83 (2.73; 52.46)	99.4
	0.30	557 (521; 591)	334 (300; 370)	−4.10 (−7.76; −0.58)	0.0169 (0.00045; 0.0574)	43.34 (11.12; 65.54)	95
original ITT MA (two RCTs)		444	447	−3.00 (−4.92; −1.09)	0.0021	0	--

The REML random effect method was applied for the mean difference, in mmHg, *m*_1_–*m*_0_ in our notation above. *p_i_*: percentages of adherence in the intervention group. *p_c_*: percentages of adherence in control group *n_1_* and *n_0_*: sample sizes per protocol in intervened and control groups, respectively. * Median (P_2.5_; P_97.75_) per protocol. ** Median (95% Confidence Interval). I^2^: percentage of heterogeneity of the simulated studies per protocol.

**Table 2:** Impact of different adherence scenarios of consuming FV on DBP.

Adherence		n_1_ *	n_0_ *	Difference **	*p*-Value *	I^2^ *	Prop of *p* < 0.05
$p_{i}$	$p_{c}$						
0.70	0.10	356 (317; 394)	535 (497; 574)	−0.90 (−3.82; 1.77)	0.5168 (0.4753; 0.5869)	77.80 (71.41; 83.92)	0
	0.20	400 (362; 441)	491 (450; 529)	−1.03 (−4.31; 2.59)	0.5402 (0.4768; 0.6332)	84.73 (77.95; 90.20)	0
	0.30	446 (400; 488)	445 (403; 491)	−1.22 (−5.99; 3.27)	0.5581 (0.4721; 0.6914)	89.87 (84.41; 94.68)	0
0.80	0.10	400 (365; 437)	491 (454; 526)	−0.83 (−3.18; −1.62)	0.4842 (0.4523; 0.5328)	68.28(62.82; 76.48)	0
	0.20	444 (407; 485)	447 (406; 484)	−0.91 (−3.79; 1.85)	0.5097 (0.4653; 0.5743)	77.00 (70.25; 84.01)	0
	0.30	489 (448; 532)	402 (359; 443)	−1.05 (−4.80; 2.40)	0.5291 (0.4731; 0.6208)	82.23 (76.66; 89.40)	0
0.90	0.10	444 (414; 474)	447 (417, 477)	−0.79 (−2.93; 1.30)	0.4441 (0.4203; 0.4825)	58.89 (52.70; 66.01)	0
	0.20	489 (455; 523)	403 (368; 436)	−0.86 (−3.09; 1.53)	0.4707 (0.4412; 0.5166)	67.36 (60.38; 74.73)	0
	0.30	534 (496; 571)	358 (320; 395)	−0.95 (−3.79; 1.98)	0.4933 (0.4568; 0.5495)	74.39 (67.40; 81.81)	0
0.95	0.10	467 (441; 493)	424 (398, 450)	−0.78 (−2.63; 1.22)	0.4201 (0.4000; 0.4513)	52.90 (47.58; 59.61)	0
	0.20	512 (480; 544)	379 (347; 411)	−0.84 (−2.97; 1.50)	0.4477 (0.4252; 0.4827)	61.66 (54.96; 69.19)	0
	0.30	557 (521; 591)	334 (300; 370)	−0.92 (−3.63; 1.82)	0.4711 (0.4448; 0.5107)	69.01 (61.61; 76.35)	0
original ITT MA (two RCTs)		444	447	−0.74 * (−2.30; +0.83)	0.3580	36.02	--

The REML random effect method was applied for the mean difference, in mmHg, *m*_1_–*m*_0_ in our notation above. *p_i_*: percentages of adherence in the intervention group. *p_c_*: percentages of adherence in control group. *n*_1_ and *n*_0_: sample sizes per protocol in intervened and control groups, respectively. * Median (P_2.5_; P_97.75_) per protocol. ** Median (95% Confidence Interval). I^2^: percentage of heterogeneity of the simulated studies per protocol.

**Table 3:** Impact of different adherence scenarios of consuming FV on LDL.

Adherence		n_1_ *	n_0_ *	Difference **	*p*-Value *	I^2^ *	Prop of *p* < 0.05
$p_{i}$	$p_{c}$						
0.70	0.10	100 (89; 111)	151 (140; 162)	−0.28 (−0.52; −0.07)	0.0099 (0.0026; 0.0209)	0 (0; 0) †	100
	0.20	113 (101; 125)	138 (126; 150)	−0.34 (−0.57; −0.14)	0.00138 (0.00005; 0.0075)	0 (0; 8.54) †	99.9
	0.30	125 (112; 137)	126 (114; 139)	−0.44 (−0.65; −0.26)	0.00004 (<0.00001; 0.0156)	0 (0; 67.90) †	99.9
0.80	0.10	113 (102; 123)	138 (128; 149)	−0.24 (−0.46; −0.05)	0.0223 (0.0097; 0.0367)	0 (0; 0)	100
	0.20	125 (114; 137)	126 (114; 137)	−0.29 (−0.50; −0.11)	0.00602 (0.0012; 0.0152)	0 (0; 0)	100
	0.30	138 (125; 149)	113 (102; 126)	−0.34 (−0.56; −0.15)	0.00073 (0.000013; 0.0055)	0 (0; 0)	100
0.90	0.10	125 (117; 134)	126 (117; 134)	−0.21 (−0.42; −0.01)	0.0408 (0.0248; 0.0575)	0 (0; 0)	85.1
	0.20	137 (127; 147)	114 (104; 124)	−0.24 (−0.45; −0.05)	0.0177 (0.0067; 0.0322)	0 (0; 0)	100
	0.30	150 (139; 161)	101 (90; 112)	−0.29 (−0.48; −0.10)	0.0049 (0.0006; 0.0141)	0 (0; 0) †	100
0.95	0.10	131 (124; 139)	120 (112; 127)	−0.20 (−0.41; 0.02)	0.0526 (0.0349; 0.0683)	0 (0; 0)	36.9
	0.20	144 (135; 153)	107 (98; 116)	−0.23 (−0.42; −0.03)	0.0266 (0.0132; 0.0422)	0 (0; 0)	99.4
	0.30	156 (147; 167)	95 (84; 104)	−0.26 (−0.48; −0.06)	0.0095 (0.0024; 0.0208)	0 (0; 0) †	100
original ITT MA (two RCTs)		125	126	−0.17 ** (−0.38; 0.03)	0.1026	0	---

The fixed effect method was applied for the mean difference, in mmol/L, *m*_1_–*m*_0_ in our notation above. *p_i_*: percentages of adherence in the intervention group. *p_c_*: percentages of adherence in control group. *n_1_* and *n_0_*: sample sizes per protocol in intervened and control groups, respectively. * Median (P_2.5_; P_97.75_) per protocol. ** Median (95% Confidence Interval). I^2^: percentage of heterogeneity of the simulated studies per protocol. † Given that at least one case presented I^2^ > 25%, REML was simulated with random effect.

**Table 4:** Impact of different adherence scenarios of consuming VF on HDL.

Adherence		n_1_ *	n_0_ *	Difference **	*p*-Value *	I^2^ *	Prop of *p* < 0.05
$p_{i}$	$p_{c}$						
0.70	0.10	100 (89; 111)	151 (140; 162)	−0.02 (−0.023; −0.018)	0.6702 (0.6194; 0.7040)	0 (0; 0)	0
	0.20	113 (101; 125)	138 (126; 150)	−0.02 (−0.030; −0.021)	0.6016 (0.5146; 0.6548)	0 (0; 0)	0
	0.30	125 (112; 137)	126 (114; 139)	−0.03 (−0.042; −0.026)	0.5051 (0.3710; 0.5848)	0 (0; 0)	0
0.80	0.10	113 (102; 123)	138 (128; 149)	−0.02 (−0.019; 0.016)	0.7087 (0.6748; 0.7323)	0 (0; 0)	0
	0.20	125 (114; 137)	126 (114; 137)	−0.021 (−0.024; −0.018)	0.6562 (0.6089; 0.6942)	0 (0; 0)	0
	0.30	138 (125; 149)	113 (102; 126)	−0.03 (−0.031; −0.02)	0.5911 (0.5105; 0.6489)	0 (0; 0)	0
0.90	0.10	125 (117; 134)	126 (117; 134)	−0.02 (−0.017; −0.015)	0.7406 (0.7188; 0.7502)	0 (0; 0)	0
	0.20	137 (127; 147)	114 (104; 124)	−0.02 (−0.020; −0.016)	0.7030 (0.6689; 0.7299)	0 (0; 0)	0
	0.30	150 (139; 161)	101 (90; 112)	−0.02 (−0.025; −0.019)	0.6550 (0.6019; 0.6933)	0 (0; 0)	0
0.95	0.10	131 (124; 139)	120 (112; 127)	−0.01 (−0.016; −0.014)	0.7544 (0.7377; 0.7671)	0 (0; 0)	0
	0.20	144 (135; 153)	107 (98; 116)	−0.02 (−0.017; −0.016)	0.7229 (0.6958; 0.7425)	0 (0; 0)	0
	0.30	156 (147; 167)	95 (84; 104)	−0.02 (−0.047; −0.050)	0.6824 (0.6429; 0.7103)	0 (0; 0)	0
original ITT MA (two RCTs)		125	126	−0.01 * (−0.10; 0.08)	0.7907	0	---

The fixed effect method was applied for the mean difference, in mmol/L, *m*_1_–*m*_0_ in our notation above. *p_i_*: probability of adhering to treatment. *p_c_*: probability of consuming VF in control group. *n_1_* and *n_0_*: sum of the true sample sizes in the intervention and control groups, respectively. The values represent: Median (P_2.5_; P_97.75_). I^2^: percentage of heterogeneity. * 95% confidence interval.

## Data Availability

The data presented in this study are available on request from the corresponding author.

## Supplementary Materials

The following are available online at https://www.mdpi.com/article/10.3390/nu13072352/s1, Computer Code S1: Commands to deduce analysis per protocol with data known by intention to treat.
