# Supplementary material for: A Method to Estimate the Efficacy vs. Effectiveness in Meta-Analysis of Clinical Trials with Different Adherence Scenarios: A Monte Carlo Simulation Study in Nutrition
Source: Nutrients. 2021 Jul 9;13(7):2352. doi: 10.3390/nu13072352 (PMC8308700; doi:10.3390/nu13072352)
Supplement: Supplementary file 1 [file nutrients-13-02352-s001.zip › nutrients-1244954-supplementary.pdf]

Computer Code S1: Commands to deduce analysis per protocol with data known by intention to treat.

```
###
##
# COCHRANE STUDY OF FV AND CARDIOVASCULAR DISEASE MARKERS
##
###


## ------------- PAS ------------

# Data frame with primary studies

ds <- data.frame(
  id=c(1,2), ## ID study
  m.i=c(-2, -1.6),   ## arithmetic mean of SBP in the intervention group
  m.c=c(1.4, -.6),   ## arithmetic mean of SBP in the control group
  s.i=c(13.5, 16.4), ## standard deviation in the intervention group
  s.c=c(14.6, 17.6), ## standard deviation in the control group
  n.i=c(344, 100),   ## sample size in the intervention group
  n.c=c(346, 101)    ## sample size in the control group
)

ds
library(metafor)

met1 <- escalc(measure = "MD", m1i=m.i, sd1i=s.i, n1i=n.i, m2i=m.c,
sd2i=s.c, n2i=n.c, data=ds)
met1
meta.rma1 <- rma(yi,vi, method = "REML", data=met1)
meta.rma1


## ------- COMMANDS TO DEDUCT SIMULATIONS of PER PROTOCOL STATISTICS ----
----

set.seed(12345)

s <- 1000

dm <- data.frame(sim=c(1:s))

for(x in 1:s){ ## Loop through the 1000 simulations

      # simulate adherence in each study

  ds$p.i <- rbeta(nrow(ds), 70, 30)  # In the intervention group pi=0.70
(values are changed for each planned scenario)

  ds$p.c <- rbeta(nrow(ds), 10, 90)  # In the control group pc=0.30
(values are changed for each planned scenario)
```

```
## Estimation of the means and variances of effect in those who truly
take FV and those who do not

## In each study separately

for(r in 1:nrow(ds)){

# Data observed in allocation groups: intervention (i) and control(c)

    MO <- c(ds$m.i[r], ds$m.c[r])      # Medias
    VO <- c(ds$s.i[r]^2, ds$s.c[r]^2) # Varianzas
    NO <- c(ds$n.i[r], ds$n.c[r])      # muestra

# Adherence probability matrix
    PM <- matrix(c(ds$p.i[r], ds$p.c[r], 1-ds$p.i[r], 1-ds$p.c[r]),
nrow=2)

# Estimated data in the real fruit groups: In those who take (1) and do
not take (0)
    MT <- solve(PM, MO)            # mean
    ST <-(solve(PM, VO + MO^2))    # Variances (intermediate step)
    VT=ST-MT^2                     # Variances
    NT <- t(PM) %*% NO             # sample sizes

# Put the results in the database
    ds$m.1[r] <- MT[1] # mean in those who truly take FV
    ds$m.0[r] <- MT[2] # mean in which they do not really eat FV
    ds$s.1[r] <- sqrt(VT[1]) # standard deviation in those who truly eat
                  fruit
    ds$s.0[r] <- sqrt(VT[2]) # standard deviation in which they do not
                  really eat FV
    ds$n.1[r] <- round(NT[1]) # sample size of those who truly eat FV
                  ds$n.0[r] <- round(NT[2]) # sample size of those who
                  truly do not eat FV
      }
   ds

## Obtain 1000 meta-analyzes with data per protocol

  met2<-escalc(measure = "MD", m1i=m.1, sd1i=s.1, n1i=n.1, m2i=m.0,
sd2i=s.0, n2i=n.0, data=ds)

  meta.rma2<-rma(yi,vi, method="REML", data=met2) # calculates meta-
analysis with declared intake


# extract the data from the 1000 simulations with pi=0.70 and pc=0.30

  dm[x, "media_70_10"]<- meta.rma2$beta
  dm[x, "se_70_10"]<-meta.rma2$se
  dm[x, "p-valor_70_10"]<- meta.rma2$pval
  dm[x, "inf_70_10"]<- meta.rma2$ci.lb
  dm[x, "sup_70_10"]<- meta.rma2$ci.ub
  dm[x, "n_1_70_10"]<- sum(n_1)
  dm[x, "n_0_70_10"]<- sum(n_0)
  dm[x, "i2_70_10"]<- meta.rma2$I2
}
```

```
PAS_70_30_REML<-data.frame(dm)  ## The database is generated with
simulations per protocol for the scenario pi = 0.70 and pc = 0.30. The
process is repeated for the remaining 11 adherence scenarios.
```
